# Partial trisomy 4q and monosomy 5p inherited from a maternal translocationt(4;5)(q33; p15) in three adverse pregnancies

**DOI:** 10.1186/s13039-020-00492-4

**Published:** 2020-06-30

**Authors:** Jingbo Zhang, Bei Zhang, Tong Liu, Huihui Xie, Jingfang Zhai

**Affiliations:** Department of Prenatal Diagnosis Medical Center of Xuzhou Central Hospital, Xuzhou Clinical Schools of Xuzhou Medical University and Nanjing Medical University, 199 South Jiefang Road, Xuzhou, 221009 Jiangsu China

**Keywords:** Balanced translocation, partial trisomy 4q, monosomy 5p, Cri-du-Chat syndrome

## Abstract

**Background:**

Carriers of balanced reciprocal chromosomal translocations are at known reproductive risk for offspring with unbalanced genotypes and resultantly abnormal phenotypes. Once fertilization of a balanced translocation gamete with a normal gamete, the partial monosomy or partial trisomy embryo will undergo abortion, fetal arrest or fetal malformations. We reported a woman with chromosomal balanced translocation who had two adverse pregnancies. Prenatal diagnosis was made for her third pregnancy to provide genetic counseling and guide her fertility.

**Case presentation:**

We presented a woman with chromosomal balanced translocation who had three adverse pregnancies. Routine G banding and CNV-seq were used to analyze the chromosome karyotypes and copy number variants of amniotic fluid cells and peripheral blood. The karyotype of the woman was 46,XX,t(4;5)(q33;p15). During her first pregnancy, odinopoeia was performed due to fetal edema and abdominal fluid. The umbilical cord tissue of the fetus was examined by CNV-seq. The results showed a genomic gain of 24.18 Mb at 4q32.3-q35.2 and a genomic deletion of 10.84 Mb at 5p15.2-p15.33 and 2.36 Mb at 15q11.1-q11.2. During her second pregnancy, she did not receive a prenatal diagnosis because a routine prenatal ultrasound examination found no abnormalities. In 2016, she gave birth to a boy. The karyotype the of the boy was 46,XY,der(5)t(4;5)(q33;p15)mat. The results of CNV-seq showed a deletion of short arm of chromosome 5 capturing regions 5p15.2-p15.33, a copy gain of the distal region of chromosome 4 at segment 4q32.3q35.2, a duplication of chromosome 1 at segment 1q41q42.11 and a duplication of chromosome 17 at segment 17p12. During her third pregnancy, she underwent amniocentesis at 17 weeks of gestation. Chromosome karyotype hinted 46,XY,der(5)t(4;5)(q33;p15)mat. Results of CNV-seq showed a deletion of short arm (p) of chromosome 5 at the segment 5p15.2p15.33 and a duplication of the distal region of chromosome 4 at segment 4q32.3q35.2.

**Conclusions:**

Chromosomal abnormalities in three pregnancies were inherited from the mother. Preimplantation genetic diagnosis is recommended to prevent the birth of children with chromosomal abnormalities.

## Background

Balanced reciprocal chromosomal translocations (RCT) involve the exchange of chromosomal material between the arms of heterologous chromosomes, thus changing genetic location rather than the amount of genetic material. The balanced chromosome carrier is healthy but at a high risk of having a chromosomally unbalanced offspring. Parents with balanced translocations may transmit unbalanced chromosomes to their children, usually resulting in partial monosomy and partial trisomy. The degree of clinical expression of segmental aneuploidy often varies with the size of chromosomal region involved [1].

5p deletion syndrome, also known as Cri-du-Chat syndrome, is a rare cytogenetic condition, caused by variable size deletions in the short arm of chromosome 5. The main clinical features at birth are high-pitched cat-like cry. Other notable characteristics are developmental delay, severe psychomotor and mental retardation, rounded face, hypertelorism, epicanthal folds, broad nasal bridge, short neck and micrognathia, low-set ears, abnormal dermatoglyphics, hypotonia and down-turned corners of the mouth [2, 3].

Duplication/trisomy of 4q has been reported in several patients since it was first described in 1972 [4], most of which resulted from the malsegregation of a familial translocation. Usually, the phenotype is complicated by concomitant monosomy of another chromosome segment [5, 6]. The most common clinical features of trisomy 4q include developmental delay, mild-to-severeintellectual disability, growth deficiency, microcephaly, broad/prominent nasal bridge, prominent/low-set ears, epicanthic folds, hypertelorism and short neck.

We described a pregnant woman with a balanced translocation who had two adverse pregnancies. In the case, the three pregnancies of the woman had similar genotypes and CNVs, and the defects showed a certain similarity. Her three pregnancies were characterized by 4q duplication and 5p deletion. Prenatal diagnosis was made for her third pregnancy to provide genetic counseling and guide her fertility.

## Case presentation

A 33-year-old, gravida 3, para 1, woman who had a history of two adverse pregnancies, was referred to our center at 17 weeks of gestation. Her first pregnancy was in 2014. At 16 weeks of her first pregnancy, ultrasound showed fetal edema and ascites. Then, odinopoeia was performed. The umbilical cord tissue of the lost fetus was examined by CNV-seq technique and the results indicated a genomic gain of 24.18 Mb at 4q32.3-q35.2 and a genomic deletion of 10.84 Mb at 5p15.2-p15.33 and a genomic deletion of 2.36 Mb at15q11.1-q11.2. The deleted region of chromosome 15 happened de novo and was a polymorphism. Unfortunately, we were unable to show the picture of this results in our report due to the update of the computer system. During the woman’s second pregnancy, no abnormalities were found on routine prenatal examinations. And she did not make a prenatal diagnosis. Sadly, her son, born in 2016, suffered from Cri-du-Chat syndrome. The main clinical features of her son at birth were plaintive high-pitched monochromatic cry similar to the mewing of a cat and low-set ears. Her son is now more than 3 years old and shows significant developmental delays, including speech and movement. This is her third pregnancy. And, there were no significant complications or exposures during this pregnancy. Routine prenatal ultrasound was normal. The phenotype of the woman was normal. They were in a non-consanguineous marriage. There was no family history of miscarriage, congenital anomalies, or infertility determined from either the husband or the wife. Her husband has a normal karyotype. Amniotic fluid of the fetus and peripheral blood of the woman and her son were collected for karyotype analysis and genome-wide copy number variants (CNVs) after informed consent. This study was approved by the ethics committee.

## Materials and methods

### Chromosome G-banding

Chromosome analysis was performed on G-band metaphases prepared from amniotic fluid samples and cultured peripheral blood lymphocytes according to the laboratory’s standard protocols. Twenty metaphases were analyzed for each individual sample.

### Copy-number variation sequencing (CNV-seq)

50 ng of DNA was fragmented, and DNA libraries were constructed by end filling, adapter ligation and polymerase chain reaction (PCR) amplification. DNA libraries were subjected to massively parallel sequencing on the NextSeq 500 platform (Illumina, San Diego, CA, USA) to generate approximately 5 million raw sequencing reads with 36-base pair (bp) genomic DNA sequences [7, 8]. The results of CNVs were identified and mapped by referring to the hg19 version of the human genome and the publicly available databases, including Decipher, Database of Genomic Variants (DGV), 1000 genomes, and Online Mendelian Inheritance in Man (OMIM), PubMed and other public databases available. Chromosome profiles were finally plotted as copy number (Y-axis) vs 20-kb count windows (X-axis). A blue line was used to indicate the mean copy number across each chromosome to identify the nature and map position of any deleted or duplicated regions.

## Results

**Fig. 1:**
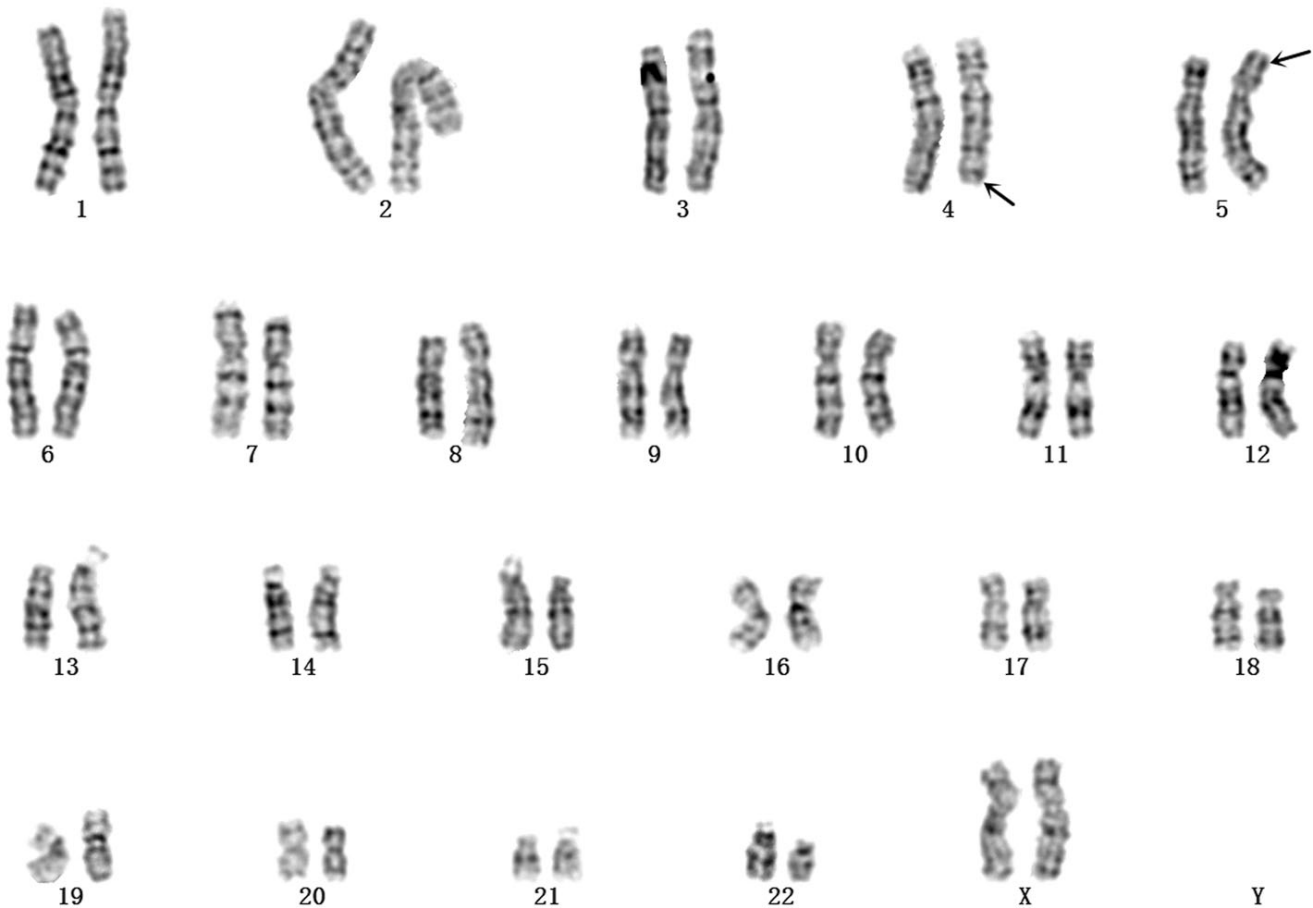
The karyotype of the women showed a balanced translocation between chromosomes 4 and 5:46,XX,t(4;5)(q33;p15)

**Fig. 2:**
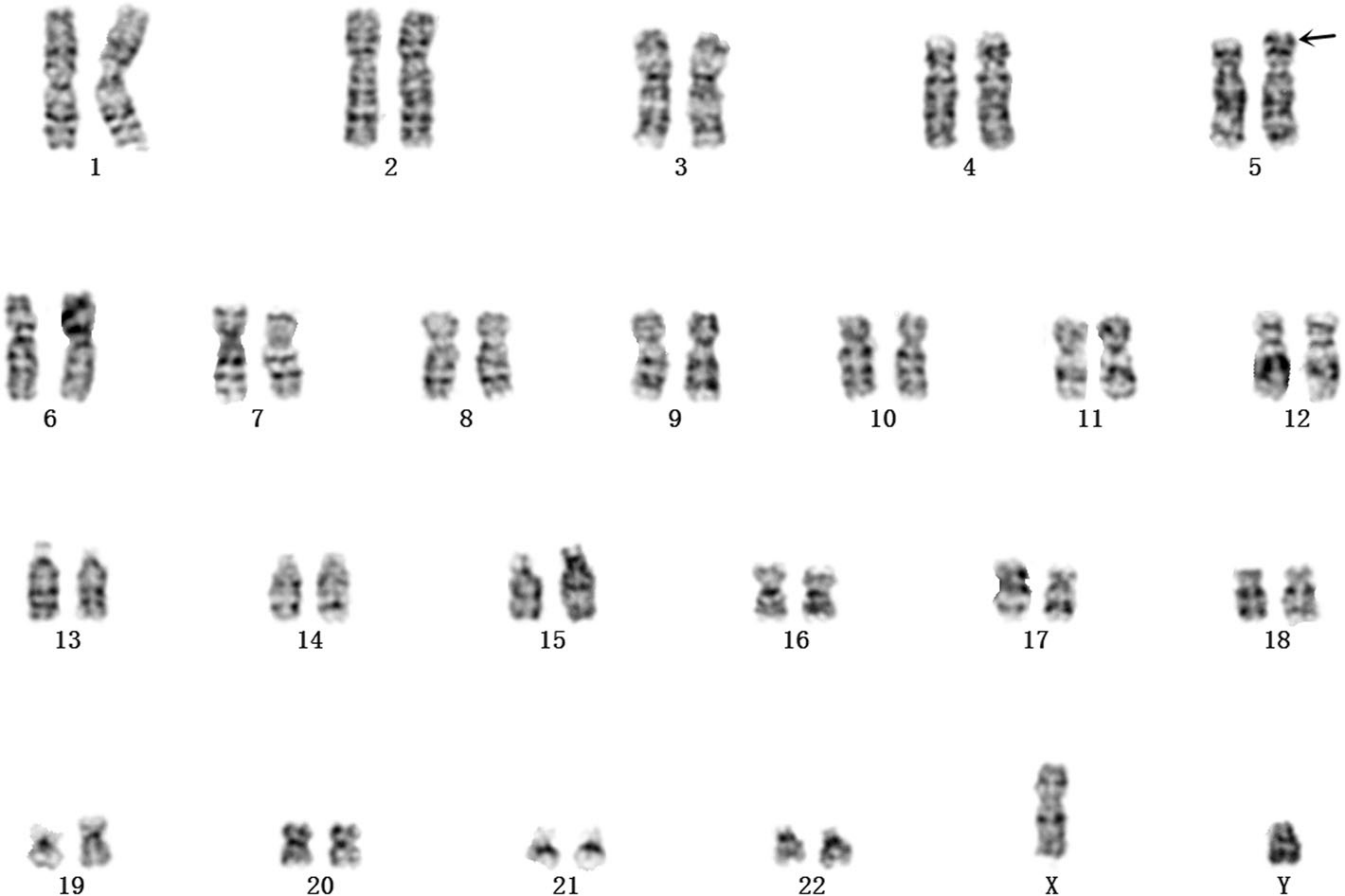
The karyotypethe of the boy is 46,XY,der(5)t(4;5)(q33;p15)mat at the level of 300 ~ 400 bands

**Fig. 3:**
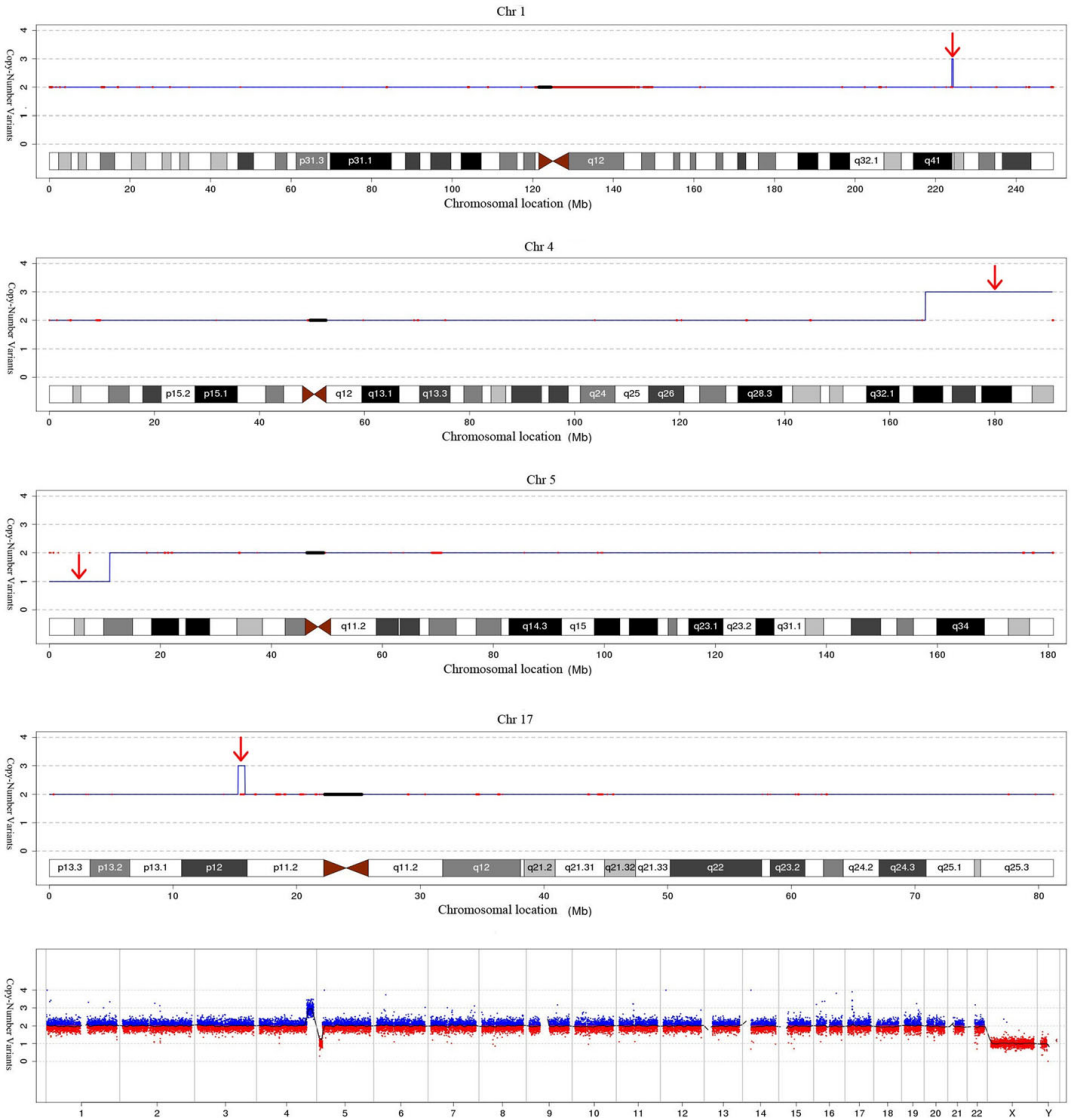
The results of CNV-seq analysis. A deletion of short arm (p) of chromosome 5 from position 20001 to position 10880000 capturing regions 5p15.2p15.33. A copy gain of the distal region of chromosome 4 at segment 4q32.3q35.2 from position 166780001 to position 190940000 and a duplication of chromosome 1 at segment 1q41q42.11 from position 224040001 to position 224380000 and a duplication of chromosome 17 at segment 7p12 from position 15260001 to position 15820000

**Fig. 4:**
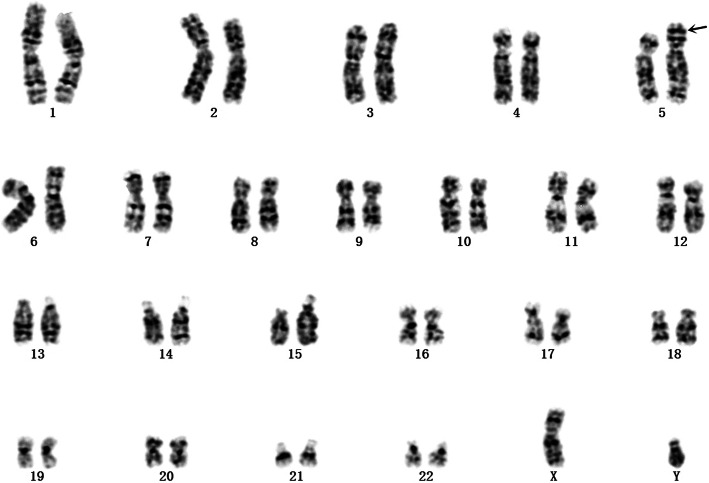
The karyotype of the third pregnancy showed 46,XY,der(5)t(4;5)(q33;p15)mat at the level of 300 ~ 400 bands

**Fig. 5:**
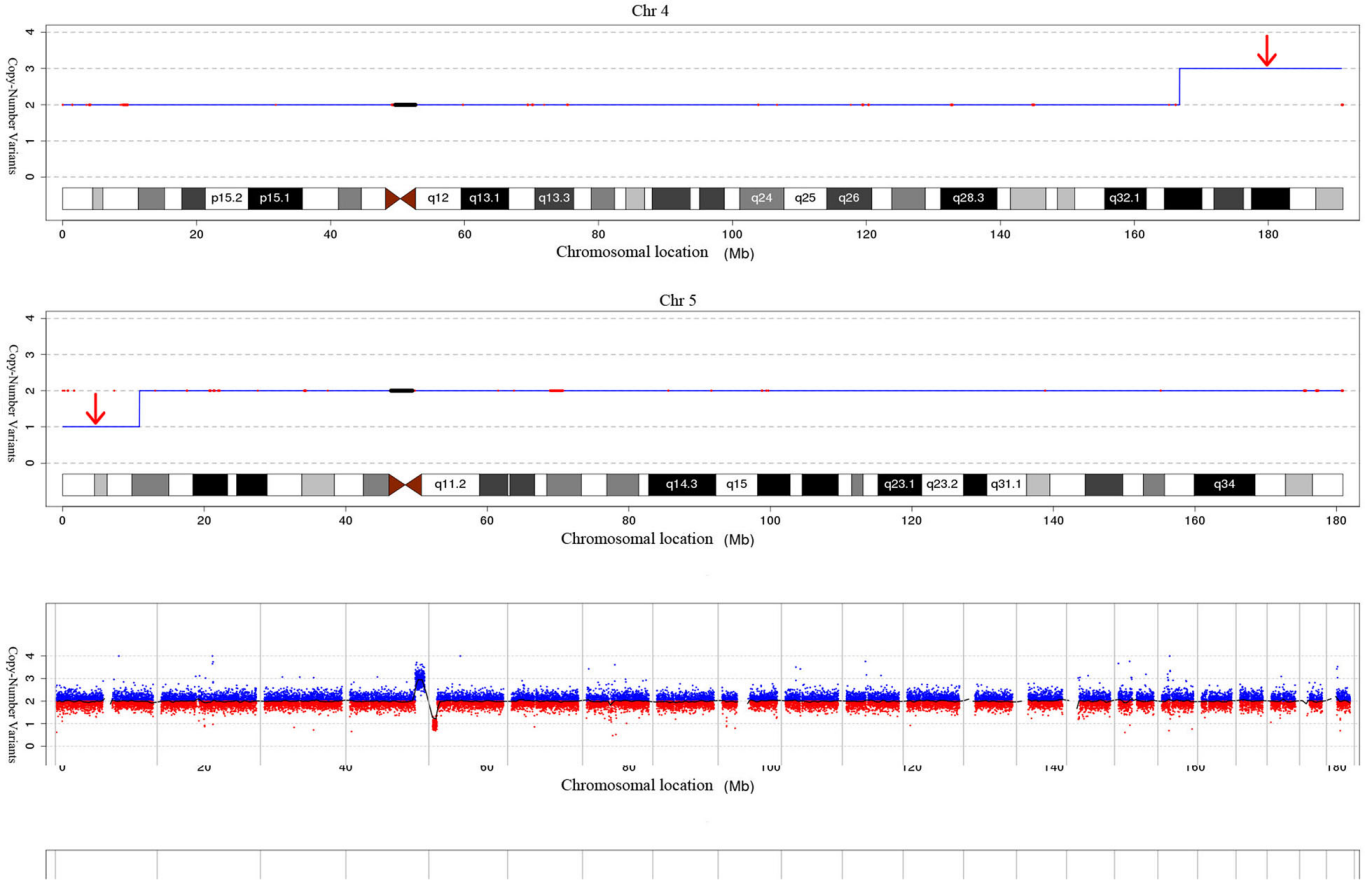
A CNV-seq analysis of the amniotic fluid cells showing a deletion at 5p15.2p15.33 (chr5:g.20001_10860000) and a copy gain at 4q32.3-q35.2(chr4:166760001–190940000)

Analyse of the woman’s peripheral blood lymphocytes showed a balanced translocation between chromosomes 4 and 5:46,XX,t(4;5)(q33;p15) (Fig. 1). The boy was referred to the prenatal diagnostic laboratory at Xuzhou Central Hospital affiliated with Xuzhou Medical University, for cytogenetic analysis. Chromosomal studies were performed on the basis of G-banding technique. The results indicated that the karyotypethe of the boy was 46,XY,der(5)t(4;5)(q33;p15)mat (Fig. 2). Further analysis of the structural chromosomal rearrangement in the boy was done using CNV-seq technique on interphase chromosomes. The results showed a deletion at 5p15.2p15.33 from position 20001 to position 10880000. And the results depicted a copy gain of the distal region of chromosome 4 at segment 4q32.3q35.2 from position 166780001 to position 190940000, meanwhile a 0.34-Mb duplication at 1q41q42.11 from position 224040001 to 224380000 and a 0.56-Mb duplication at 17p12 from position 15260001 to 15820000 were found unexpectedly (Fig. 3). However, no clear pathogenic information related to the fragments of Chromosome 1 and 17 has been found in public database resources to date. Amniocentesis was performed at 17 weeks of gestation for her third pregnancy. The fetal karyotype hinted 46,XY,der(5)t(4;5)(q33;p15)mat (Fig. 4). The chromosomal aberrations were further detected by CNV-seq, and the results showed a deletion at 5p15.2p15.33 from position 20001 to position 10860000. And the results depicted a duplication at 4q32.3q35.2 from position 166760001 to position 190940000 (Fig. 5 and Table 1).

**Table 1:** Karyotype and CNVs results of the woman’s three pregnancies

The number of pregnancy	phenotypic	Sonographic findings	karyotype	results of CNV-seq	Pregnancy outcomes	gender
First pregnancy	/	fetal edema and fluid buildup in the abdomen	/	dup(4)(q32.3q35.2)	odinopoeia	male
				del(5)(p15.33p15.2)		
				del(15)(q11.1q11.2)		
Second pregnancy	high-pitched cat-like cry, developmental delay, severe psychomotor and mental retardation	No abnormality was found on ultrasound	46,XY,der(5)t(4;5)(q33;p15)mat	del (5) (p15.33p15.2)	Born at term/boy	male
				dup (4) (q32.3q35.2)		
				dup(1)(q41q42.11)		
				dup(17)(p12)		
Third pregnancy	/	No abnormality was found on ultrasound	46,XY,der(5)t(4;5)(q33;p15)mat	del (5) (p15.33p15.2)	odinopoeia	male
				dup (4) (q32.3q35.2)

## Discussion and conclusion

Balanced translocation carriers account for 0.08–0.3% of the normal population [9]. Balanced translocation is a common type of chromosomal abnormality. The carrier status of balanced translocation is associated with recurrent miscarriage and adverse pregnancy [10]. An obvious balanced translocation may result in a clinical phenotype by gene disruption or changed expression of genes in or around the breakpoint region [11]. Several researchers have proposed three hypotheses that including a break in a gene, positional effect, and cryptic deletion or duplication to explain such phenotype abnormalities [12].

Eggs from women with balanced translocations can show several types of nuclear contents: normal, balanced or unbalanced karyotype. For carriers of balanced translocations, the normal divalent structure of the germ cell in the first meiotic division will be replaced by a trivalent (non-homologous robertsonian translocation), a tetravalent (reciprocal), or a monovalent (homologous robertsonian translocation) [13, 14], thus allowing the production of gametes of various types with different chromosomal components. The balanced translocation carrier of two chromosomes can form a quadrivalent in meiosis, which can produce various gametes according to the different ways of segregation. There are three ways of segregation: 2:2(alternate segregation, adjacent-1 segregation and adjacent-2 segregation), 3:1, and 4:0. Taking into account the exchange of these three ways, a total of 36 gametes can be produced in theory. Studies have shown that the way of segregation of balanced translocation carriers is mainly 2:2, while 3:1 and 4:0 are rare [13, 14]. Due to the diversity and randomness of gametes’ chromosome composition, the pregnancy outcomes of balanced translocation carriers vary greatly, not only among different translocation types, but also among the pregnancies of each carrier. In this case, the woman’s three pregnancies had similar genotypes and CNV, indicating that the pregnancy outcome of the balanced translocation carrier may occur repeatedly. This suggested that the similarity of the abnormal karyotype and the preference for a certain abnormal product formed in meiosis may be related to the location of the fracture point [14, 15].

Distal deletions of 5p cause Cri-du-Chat syndrome (OMIM #123450) with a cat-like cry in infancy, dysmorphic facial features, microcephaly and intellectual disability. In the present case, clinical features of the woman’s son were characterized a high-pitched cat-like cry, developmental delay, severe psychomotor and mental retardation, which were consistent with Cri-du-Chat syndrome. Most 5p deletions are caused by new mutations, of which 80% ~ 90% come from the paternal line and are caused by chromosome breakage during gametogenesis [16]. In the case, the 5p terminal deletion was consistent in the woman’s three pregnancies. The father of the fetus has a normal karyotype, so it is considered that the 5p deletion of the three fetuses are inherited from the mother’s balanced translocation. This case of maternal inheritance of 5p deletion syndrome is clinically rare. The size of the deletion associated with 5p deletion syndrome ranges from 5 to 40 Mb [17]. The larger the deleted fragment, the more severe the patient’s clinical symptoms. The clinical phenotype may be related to genes deleted on the short arm of chromosome 5. Surprisingly, the woman’s three pregnancies we reported suggested partial deletion of short arm of chromosome 5. The results of the three pregnancies by CNV-seq indicated that chromosome 5 had a 10.86 Mb region deletion at p15.2-p15.33. The deleted region encompasses 87% candidate genes of the Cri du Chat Syndrome (5p deletion). The critical region for the cat-like cry was mapped to a 1 Mb interval at 5p15.32 encompassing a candidate gene *ICE1*(OMIM *617958), which regulates small nuclear RNA transcription; the *TERT* (OMIM *187270) gene at 5p15.33 and the *SEMA5A*(OMIM *609297) and *CTNND2*(OMIM *604275) genes at 5p15.31p15.2 were considered candidate genes for autistic and cognitive phenotypes [18]. This gene encodes a protein which plays a critical role in neural development, particularly in the formation and maintenance of dendritic spines and synapses [19]. The gene may be linked to the child’s developmental delay. In addition, a 0.34-Mb duplication at 1q41q42.11 and a 0.56-Mb duplication at 17p12 were found. We thought the duplication of these segment happen de novo. And no clear pathogenic information related to the fragments of Chromosome 1 and 17 has been found in public database resources to date. Therefore, the duplication regions of chrosome 1 and 17 were not associated with the child’s phenotype.

Duplication/trisomy of 4q has been reported in several patients since it was first described in 1972 [4], most of which resulted from the malsegregation of a familial translocation. Patients with dup4q syndrome have variable clinical features, which are both related to the size and gene content of duplicated segment and specific associated monosomy [20]. Usually, concomitant monosomy of another chromosome segment complicates the phenotype [5, 6]. Elghezal et al. showed that segment 4q35 was probably involved in microcephaly, severe growth and mental retardation [21]. In this case, there was a 24.16-Mb duplication at 4q32.3-q35.2 in the woman’s three pregnacies. After a public database query, one case of 4q35 duplication was reported in the literature. The main clinical features are focal segmental glomerulosclerosis, bilateral sensorineural hearing loss, bilateral retinopathy, basal ganglia calcification, reflex myoclonus, retardation, etc. [PMID: 20191367]. In this case, all the CNV-seq results of the three pregnancies indicated a 24.16-Mb duplication of at 4q32.3q35.2. According to OMIM database, several pathogenic genes were contained at 4q32.3q35.2 including *PALLD, TLL1, NEK1, HPGD, VEGFC, AGA* and *TENM3* and so on. These genes are associated with familial pancreatic cancer, short-rib thoracic dysplasia, enlargement of the nail plate and terminal segments of the fingers and toes, lymphatic malformation, aspartylglucosaminuria. In this case, the child does not currently have any symptoms mentioned above, but he needs further follow-up. Because abnormal chromosomal structure can affect the normal division of germ cells, the risk of fetal malformation or miscarriage remains high in patients in the following pregnancy [22, 23]. Although ultrasound abnormalities in the second trimester and abnormalities in maternal serum markers have been reported to be associated with 5p deletion syndrome, 5p deletion syndrome has no characteristic changes in ultrasound and serum markers, so it is difficult to identify 5p deletion syndrome in the prenatal [2, 24–30]. In this case, because the ultrasound showed no abnormalities during the second pregnancy, the woman did not receive a prenatal diagnosis, resulting in some chromosomal deletions or duplications in the child. Balanced translocation couples may attempt to conceive naturally. Although the theoretical risk of spontaneous abortion is high for both carriers, many cases have produced offspring with normal phenotypes after genetic counseling in practice. Of course, prenatal diagnosis should be recommended after a natural pregnancy. From our point of view, if necessary, assisted reproductive technology and preimplantation diagnosis can be selected to reduce the risk of birth of children with abnormal chromosomes [31].

At present, for balanced translocation carriers, it is generally recommended to select preimplantation genetic diagnosis (PGD) to obtain offspring, which may be a best choice [32]. It should be noted that clinical genetic counseling should be conducted so that patients can fully understand the advantages and disadvantages of different fertility methods, as well as the risks involved. At the same time, the importance of prenatal diagnosis should be clarified and informed choices should be made.

## Abbreviations

CNV: Copy number variations
DGV: Database of Genomic Variants
OMIM: Online Mendelian Inheritance in Man
PCR: Polymerase chain reaction
PGD: Preimplantation genetic diagnosis
